# Global prevalence of iron deficiency anaemia among children aged 5–12 years: a systematic review and meta-analysis

**DOI:** 10.7189/jogh.16.04027

**Published:** 2026-01-23

**Authors:** Pattarapan Sukwuttichai, Nattapong Tidwong, Natapohn Chaipichit, Teerapon Dhippayom, Witoo Dilokthornsakul, Piyameth Dilokthornsakul

**Affiliations:** 1 Department of Pharmaceutical Care, Faculty of Pharmacy, Chiang Mai University, Chiang Mai, Thailand; 2 Pharmaceutical Care Training Center, Department of Pharmaceutical Care, Faculty of Pharmacy, Chiang Mai University, Chiang Mai, Thailand; 3 Center for Medical and Health Technology Assessment, Department of Pharmaceutical Care, Faculty of Pharmacy, Chiang Mai University, Chiang Mai, Thailand; 4 The Research Unit of Evidence Synthesis, Faculty of Pharmaceutical Sciences, Naresuan University, Phitsanulok, Thailand; 5 Department of Pharmacotherapy, University of Utah College of Pharmacy, Utah, USA

## Abstract

**Background:**

Iron deficiency anaemia (IDA) can lead to impairment of immunity, cognitive function, and poorer academic performance. Current health policies worldwide focus primarily on IDA prevention among preschoolers and women, overlooking school-aged children (aged 5–12 years) as a susceptible group. Through this systematic review and meta-analysis, we aimed to determine the prevalence of IDA among this population.

**Methods:**

We searched PubMed, Embase, CINAHL, and EBSCO Open Dissertation from inception until July 2023 for English-language observational studies reporting on the prevalence of IDA children aged 5–12. We calculated the pooled prevalence using a random-effect model and performed subgroup analyses by regions, countries’ income, and diagnostic criteria. We assessed the study quality using Hoy’s risk of bias tool.

**Results:**

We included 55 studies involving over 2.1 million children. None of these studies had a high risk of bias. The pooled global prevalence of IDA among children aged 5–12 years in community settings was 9.4% (95% confidence interval = 6.5%, 12.7%, *I^2^* = 99.6%). Subgroup analyses indicated moderate public health concerns among sub-Saharan Africa (21.9%) and South Asia (15.8%), or among low-income (29.7%) and lower-middle-income (24.5%) countries.

**Conclusions:**

IDA is an important public health issue among children aged 5–12 years globally which even poses a significant concern in some populations or regions. Our findings could guide the development of national detection strategies and health prevention programmes targeted at improving children's health and educational outcomes.

**Registration:**

PROSPERO (CRD42022335700)

Iron is a micronutrient that plays a critical role in oxygen transportation, energy production, and overall physiological growth at every stage of life. About 70% of the body’s total iron is localised in red blood cells (haemoglobin) and muscle cells (myoglobin), where it facilitates the efficient delivery and utilisation of oxygen [1]. During childhood, iron is crucial for the neurodevelopment process, such as neuronal growth, differentiation, and myelination [2]. Iron deficiency anaemia (IDA), a widespread problem in the paediatric population, is defined by a low haemoglobin concentration below age- and gender-specific normal ranges. Diagnostic criteria include plasma ferritin level <12 μg/L, transferrin saturation (TS) <10%, or, in some cases, serum ferritin values <15 μg/L. The pathogenesis of IDA arises from inadequate iron intake, limited bioavailability, or reduced iron stores, leading to insufficient haemoglobin synthesis. These deficiencies impair oxygen transport and lead to health complications, such as impaired immunity, stunted development, and cognitive function deficits [3-5].

The World Health Organization (WHO) estimates that 25% of the population worldwide is anaemic, with approximately 50% of these cases attributed to IDA [6]. It therefore classifies IDA as a public health issue when its prevalence exceeds 5% of the population, making it a significant concern worldwide [7]. Globally, IDA is most frequent in vulnerable populations, particularly infants, pre-school children, and women of childbearing age. The condition affects approximately 20% preschool children (0–4 years old) in developed countries, compared to 39% in developing countries [8]. The problem persists among older children, as well; in Indonesia, for example, the prevalence of IDA among school-aged children (6–12 years) was 32% [9]. Data from developing countries indicate no significant decline in IDA prevalence among children aged 5–14 (48.1%) compared to preschoolers (39%), highlighting the sustained burden of the condition [10].

The school age period is a stage of physical, mental, behavioural, and learning development. Children with IDA are at an increased risk of experiencing difficulties with attention, memory, and academic achievement, which may lead to long-term educational consequences [11]. Specifically, there is evidence that IDA is associated with impaired cognitive function and academic achievement among primary school children with IDA [5]. The cause of IDA among school-aged students can be attributed to factors such as low dietary iron intake, reduced intestinal iron absorption, blood loss, health conditions (parasitic diseases, infectious diseases), and socioeconomic status [8]. Research has likewise shown that children from rural areas, those from lower socioeconomic backgrounds, and those whose mothers have a lower educational level are at a higher risk for IDA [12]. The implementation of iron deficiency prevention strategies, including food-based interventions and iron supplementation, could mitigate the long-term effects of IDA in school-aged children [13].

As IDA is a preventable cause of cognitive impairment, most health policies are aimed at preschool children and women of childbearing age who exhibit the highest incidence of IDA. However, the incidence of IDA among children aged 5–12 years in some countries remains above 5%, and is reported to have had a negative impact on their learning capabilities. Although several studies have estimated the prevalence of IDA in individual countries, the overall global prevalence of IDA in children aged 5–12 years and its distribution around the world remain unknown [14–17]. Therefore, we sought to investigate the prevalence of IDA among this age group worldwide, to generate data for effective prevention and intervention strategies that would improve their nutrition and health outcomes.

## METHODS

### Search strategies

We searched PubMed, Embase, CINAHL, and EBSCO Open Dissertations from their inception to July 2023 combining terms related to children and IDA (Table S1 in **Online Supplementary Document**). We registered our protocol in PROSPERO (CRD42022335700), but amended it afterwards (Table S2 in **Online Supplementary Document**).

### Study selection

We included observational studies that reported on the prevalence of IDA among children aged 5–12 years, provided they were published in English and that they defined IDA as having both haemoglobin and serum ferritin levels below the specific cut-off values. The primary outcome was, therefore, to estimate the global prevalence of IDA among school-aged children. Haemoglobin and serum ferritin with or without transferrin saturation index were used to determine IDA.

We imported all retrieved records into Endnote, version 20.0 (Clarivate, London, UK). After deduplication, a pair of reviewers (PS, NT, NC, and WD) independently screened their titles and abstracts, followed by the full texts of any records retained after the first stage. Disagreements at both stages were resolved in discussion with a third author (PD or TD).

### Data collection and quality assessment

A pair of four reviewers (PS, NT, NC, and WD) independently extracted the following data from eligible studies using the pre-specified data collection form designed in Microsoft Excel 365 (Microsoft Corporation, Redmond, Washington, USA): author details, years of publication, study design, country of study, setting, diagnosis criteria for anaemia and IDA, number of participants, participant characteristics, prevalence of anaemia, and prevalence of IDA. Another author (PD) verified the extracted data.

### Risk of bias assessment

Pairs of four authors (PS, NT, NC, and WD) independently assessed risk of bias using Hoy’s risk of bias tool, a validated scoring system for population-based prevalence studies, to evaluate the quality of the included studies [18]. This tool examines two key domains: external validity (items 1–4) and internal validity (items 5–10). Each item is assessed as either ‘yes’ (low risk of bias) or ‘no’ (high risk of bias). Studies are categorised into three risk groups based on their total ‘yes’ scores: low risk of bias (8–10 ‘yes’ responses), moderate risk of bias (6–7 ‘yes’ responses), and high risk (0–5 ‘yes’ responses). All disagreements in the assessment between pairs were resolved through discussion among the four researchers, or by engaging another researcher (PD). Before initiating the full-scale assessment, we piloted the tool on a subset of studies to ensure the understanding and consistency among reviewers.

### Data analysis

We calculated the pooled effect size and confidence interval (CI) using a random-effect model meta-analysis. The main analysis included studies conducted in community-based settings because they reflected the prevalence of IDA in the general population, while the sensitivity analysis covered studies conducted in both community and hospital-based settings to explore the robustness of the main analysis. We determined the degree of heterogeneity using the *I^2^* statistics. We also performed subgroup analyses by publication year, region, countries’ economic status (according to the World Bank 2024 classification [19]), and diagnostic criteria to identify sources of high heterogeneity. The statistical significance threshold was set at a *P*-value of 0.05. We used Stata, version 15.0 (College Station, Texas, USA) for all analyses.

## RESULTS

The search retrieved 6747 studies, of which 312 studies were eligible for full-text review. Finally, 55 studies were included in the main analysis [20-74] (Figure S1 in the **Online Supplementary Document**).

### Characteristics of included studies

Fifty-two articles (94.5%) reported on cross-sectional studies [9,20–22,24–35,37–42,44–57,59–74], two (3.6%) on case-control studies [36,43], and one (1.8%) on a retrospective cohort study [58]. Forty-two were conducted in community settings [9,20,22,24–31,35–39,45–54,56,57,59,60,62–70,72–74], 12 in hospital settings [21,33,34,40–44,55,58,61,71], and one in both community and hospital settings [32]. Fifteen studies were carried out in Sub-Saharan Africa [20,27,29,31,33,35,36,42,47,54,56,63,66–68], 13 in East Asia and the Pacific [9,22,26,37,40,46,48,50,55,59,65,73,74], eight in South Asia [21,34,39,43,49,52,57,61], seven in Latin America [25,28,45,53,64,70,72], six in Europe and Central Asia [32,41,44,60,62,71], four in North America [24,30,58,69], and two in the Middle East and North Africa [38,51] (**Table 1**). According to counties’ incomes by the World Bank, 12 studies were conducted in high-income countries [24,26,30,32,37,38,44,58,60,62,69,71], 24 in upper-middle-income countries [9,22,25,28,33,35,36,40,41,45–47,50,51,53,55,59,64,65,68,70,72–74], 17 in lower-middle-income countries [20,21,29,31,34,39,42,43,48,49,52,54,56,57,61,63,66], and two in low-income countries [27,67].

**Table 1:** Characteristics and summary of included studies

								IDA assessment						
**Author**	**Year**	**Country of study**	**Study design**	**Setting**	**Age in years, mean (standard deviation)**	**Age range in years**	**Number of participants**	**Haemoglobin in g/dl in**	**Serum iron in μg/dl**	**Ferritin in μg/l**	**sTfR in mg/l**	**Tsat in %**	**Others**	IDA diagnosis criteria*
Abizari et al. [20]	2017	Ghana	Cross-sectional	Community	8.1 (2.1)		224	<11.5 (5–11 years), <12 (12–13 years)		<15	>8.5			WHO
Afridi et al. [21]	2017	Pakistan	Cross-sectional	Hospital		5–11	698	<11.0						Non-WHO
Al-Mekhlafi et al. [22]	2008	Malaysia	Cross-sectional	Community		7–12	241	<12.0		<10		<16%		Non-WHO
Andriastuti et al. [23]	2020	Indonesia	Cross-sectional	Community		6–9	45	<11.5		<15		<15%		WHO
Baggett et al. [30]	2006	USA	Cross-sectional	Community	9.5 (1.4)		683	<11.5		<10		<15%		Non-WHO
Cardenas et al. [24]	2005	USA	Cross-sectional	Community		6–11	870	<11.8		<12		<14%	EP	Non-WHO
Cardoso et al. [25]	2012	Brazil	Cross-sectional	Community		5–10	582	<11.5		<15	>8.3			WHO
Choi et al. [26]	2003	South Korea	Cross-sectional	Community		9–12	577	<12	<50	<12				Non-WHO
Chang Cojulun et al. [29]	2015	Kenya	Cross-sectional	Community		6–11	191	<11.5		<30		<20%		Non-WHO
Desalegn et al. [27]	2014	Ethiopia	Cross-sectional	Community	8.9 (2.01)		586	<11.5 (5–11 years), <12 (12–13 years)	<10	<15				WHO
Ferreira et al. [28]	2007	Brazil	Cross-sectional	Community		5–11	80	<11.5		<30	>8.3			Non-WHO
Fiorentino et al. [31]	2013	Senegal	Cross-sectional	Community		5–10	279	<11.5 (5–11years), <12 (12–13 years)		<15	>8.3	<16%		WHO
Gompakis et al. [32]	2006	Greece	Cross-sectional	Hospital and community		6–10	865	≤11		<10				WHO
Goosen et al. [33]	2022	South Africa	Cross-sectional	Hospital		9.5–12.3	291	<11.5 (8–11 years), <12 (12–13 years)		<15	>8.3			WHO
Gupta et al. [34]	2017	India	Cross-sectional	Hospital		5–10	132	<12		<15		<16%		WHO
Gwetu et al. [35]	2019	South Africa	cross-sectional	Community		6 - 8	184	<11.5		Yes				WHO
Hlatswayo et al. [36]	2016	South Africa	Case-control	Community		6–12	119	<11.5		<12				Non-WHO
Houghton et al. [37]	2016	New Zealand	Cross-sectional	Community	10 (0.12)		503	<11.5 (8–11 years), <12 (12–13 years)		<15	>8.3			WHO
Jaber et al. [38]	2015	Israel	Cross-sectional	Community		5–6	693	<11.5		<15				WHO
Khatiwada et al. [39]	2016	Nepal	Cross-sectional	Community	9.2 (1.9)		316	<11				<16%		Non-WHO
Khemphet et al. [40]	2022	Thailand	Cross-sectional	Hospital		5–12	88	<11.5		<15		<16%		WHO
Kilinç et al. [41]	2002	Turkey	Cross-sectional	Hospital		6–12	295	<11.5 (with MCV≤77 fL)		≤16				Non-WHO
Kuona et al. [42]	2014	Zimbabwe	Cross-sectional	Hospital		6–10	318	<11.5		<15	>8.3			Non-WHO
Liaqat et al. [43]	2022	Pakistan	Case-control	Hospital		5–12	76	NR						Non-WHO
López-Ruzafa et al. [44]	2021	Spain	Cross-sectional	Hospital		6–11	491	<11.5	Iron deficient index	<12				Non-WHO
Monárrez-Espino et al. [45]	2004	Mexico	Cross-sectional	Community		6–11	75	<11.5 (6–11 year), <12 (12–13 year)		<12		<14%		Non-WHO
Ngui et al. [46]	2012	Malaysia	Cross-sectional	Community		7–12	520	<11.5 (<12 year), <12 (12 year)		<15				WHO
Onabanjo et al. [47]	2019	South Africa	Cross-sectional	Community		7–10	556	<11.5		<12				Non-WHO
Perignon et al. [48]	2014	Cambodia	Cross-sectional	Community	9.65 (2.26)		2443	<11.5		<15	>8.3			WHO
Persson et al. [49]	1999	Bangladesh	Cross-sectional	Community		6–12	164	<11.5		<12				WHO
Porniammongkol et al. [50]	2011	Thailand	Cross-sectional	Community	10.0 (1.0)	7–11	34	<11.5		<30				WHO
Pouraram et al. [51]	2018	Iran	Cross-sectional	Community		6.0–6.9	850	<11.5		<12				WHO
Rahman et al. [52]	2015	Bangladesh	Cross-sectional	Community		6–11	94 400	<11.5		<15				WHO
Robinson et al. [53]	2018	Colombia	Cross-sectional	Community	8.5 (1.6)		3,202	<12.7		<15				WHO
Rohner et al. [54]	2007	Coˆte d’Ivoire	Cross-sectional	Community	10.2 (2.3)		281	<11.5					ZPP	WHO
Saengnipanthkul et al. [55]	2022	Thailand	Cross-sectional	Hospital		5 - 13	2 066 184	NR						WHO
Sama et al. [56]	2023	Cameroon	Cross-sectional	community	8.3 (1.7)	6–11	154	<11.5		<15				WHO
Sarna et al. [57]	2020	India	Cross-sectional	Community		5–9	2,064	<11.5		<15				WHO
Schieffer et al. [58]	2017	USA	Cohort study	Hospital		4–11	6,978	<11.8		<15				WHO
Nik Shanita et al. [59]	2018	Malaysia	Cross-sectional	Community	9.9 (0.1)	7–12	544	<11.5 (5–11 year), <12 (12–14 year)		<15				WHO
Spodaryk et al. [60]	1999	Poland	Cross-sectional	Community	Male: 11.3 (0.6), female: 11.1 (0.7)		188	<12.0 (girls), <12.2 (boys)		<12		≤16%	MCHC	Non-WHO
Sreekanth et al. [61]	2021	India	Cross-sectional	Hospital	7.1 (2.7)		52	<11.5		<30				WHO
Stellinga-Boelen et al. [62]	2007	Netherlands	Cross-sectional	Community		6–12	71	<11.0 (<6 years), <11.5 (6–12 years)		<15			MCHC	WHO
Stoltzfus et al. [63]	1997	Tanzania	Cross-sectional	Community			3254	<11		< 18	>8.3		EP	Non-WHO
Syed et al. [64]	2016	Mexico	Cross-sectional	Community	9.1 (0.1)	5–14.99	3360	<11.5 (6–11 years), <12 (12–15 years)		<15			CRP	WHO
		Columbia			9.9 (0.1)	5–14.99	8573							
Tan et al. [65]	2023	Malaysia	Cross-sectional	Community		7–11	776	<11.5		<15			CRP	WHO
Tatala et al. [66]	2004	Tanzania	Cross-sectional	Community		7–12	80	<11.5		Yes (ND)			EP	Non-WHO
Teketelew et al. [67]	2023	Ethiopia	Cross-sectional	Community		5–11	187	<11.5	<8.95	<11				Non-WHO
Turgut et al. [42]	2007	Turkey	Cross-sectional	Community	6.8 (0.2)		256	<11.0 (<6 years), <11.5 (6–12 years), <12.0 (12–14 years)		<10				Non-WHO
Valberg et al. [69]	1976	Canada	Cross-sectional	Community	7.1 (NR)	5–9	117	<11.0 (2–5 years), <11.5 (6–12 years)		<20				Non-WHO
Valencia et al. [70]	1999	Mexico	Cross-sectional	Community			296	<11.0		<12			MCHC	Non-WHO
Vendt et al. [71]	2011	Estonia	Cross-sectional	Hospital		7–12	135	<11.5		<30				Non-WHO
Villalpando et al. [72]	2015	Mexico	Cross-sectional	Community		5–11.9	4395	<11.5		<15			CRP	WHO
Yanola et al. [73]	2014	Thailand	Cross-sectional	Community		8–11	130	<11.5	< 50	<10				Non-WHO
Zheng et al. [74]	2020	China	Cross-sectional	Community	8.8 (1.4)	7–11	5295	<11.5	<10.7	<15				WHO

CRP – C-reactive protein, EP – erythrocyte protoporphyrin, IDA – iron deficiency anaemia, MCHC – mean corpuscular haemoglobin concentration, ND – not defined, NR – not reported, sTfR – soluble transferrin receptor, tsat – transferrin saturation, ZPP – zinc protoporphyrin, WHO – World Health Organization

*IDA diagnosis criteria based on haemoglobin (g/dl) and ferritin (ug/l): WHO defines IDA by haemoglobin < 11.5 g/dl (age 5–11 years) or < 12 g/dl (age 12–14 year) and ferritin <15 ug/l.

The included studies collectively enrolled 2 131 065 school-aged children, with individual study sample sizes ranging from 34 to 2 066 184. Of these children, 146 023 were assessed in community-based settings, while the remainder were evaluated in hospital-based settings. All included studies assessed IDA based on serum haemoglobin and ferritin levels. However, there were differences in blood chemistry criteria: 30 studies applied the WHO criteria [9,20,25,27,31–35,37,38,40,46,48–54,56–59,61,62,64,65,72,74], defining anaemia as haemoglobin <11.5 g/dL in children aged 5–11 years and <12 g/dL in those aged 12–14 years. The cut-off values for serum ferritin also differed across studies, ranging from 10 to 30 mcg/L. Only four studies measured C-reactive protein (CRP) levels to exclude cases with elevated serum ferritin due to infection.

### Quality assessment

According to Hoy and colleagues’ tool [18], no study had a high risk of bias, 44 studies had low risk of bias [22,24,25,27–32,34–39,41,42,44,46–59,61–65,67,69–74], while eleven had moderate risk of bias [9,20,21,26,33,40,43,45,60,66,68] (Table S3 in **Online Supplementary Document**). Studies classified as having a moderate risk of bias primarily exhibited concerns related to internal validity. Specifically, they often involved a target population that did not represent the national population, employed sampling frames that inadequately reflected the target population, utilised potentially biased sampling selection methods, and faced issues with non-response bias.

### Prevalence of IDA among school-aged children

**Figure 1:**
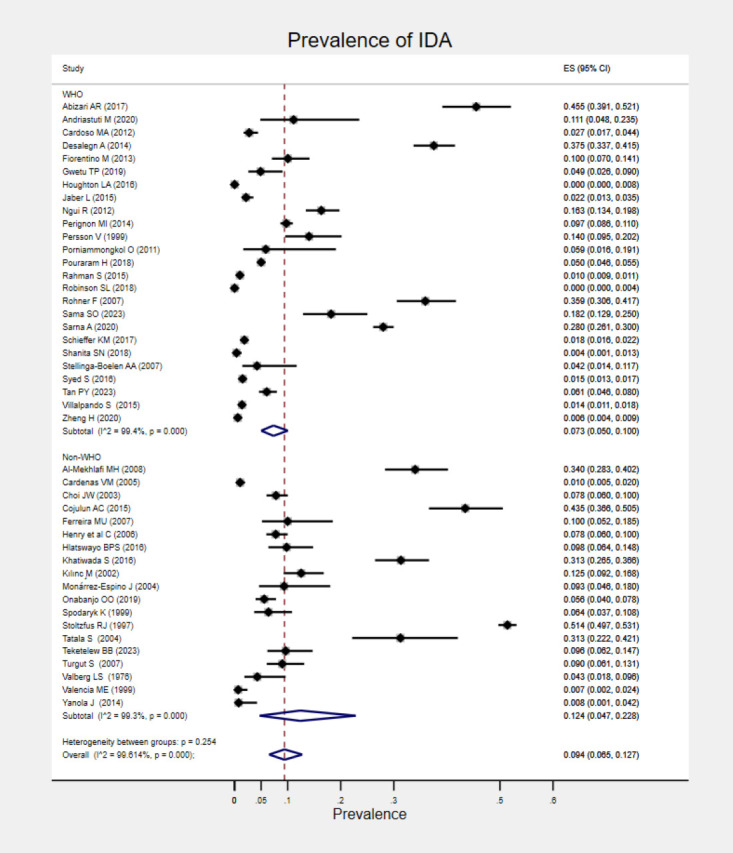
Pooled prevalence of IDA among school-aged children in community settings classified by IDA assessment criteria.

The prevalence of IDA reported in each study is presented in Table S4 in the **Online Supplementary Document**. The global pooled prevalence of IDA was 9.4% (95% CI = 6.5–12.7, *I^2^* = 99.6%). The pooled prevalence of IDA from studies published before 2015 was 11.7% (95% CI = 5.5–19.7, *I^2^* = 99.3%), compared to 7.0% (95% CI = 4.7–9.6, *I^2^* = 99.4%) in studies published in 2015 or later. Due to variations in the criteria used for IDA assessment, a subgroup analysis was conducted based on the haemoglobin cut-off values. According to the WHO diagnostic criteria of IDA, the pooled prevalence of IDA was 7.3% (95% CI = 5.0–10.0, *I^2^* = 99.4%). In contrast, studies utilising non-WHO criteria reported a higher prevalence of 12.4% (95% CI =4.7–22.8, *I^2^* = 99.3%) (**Figure 1**).

### Sensitivity and subgroup analyses

**Figure 2:**
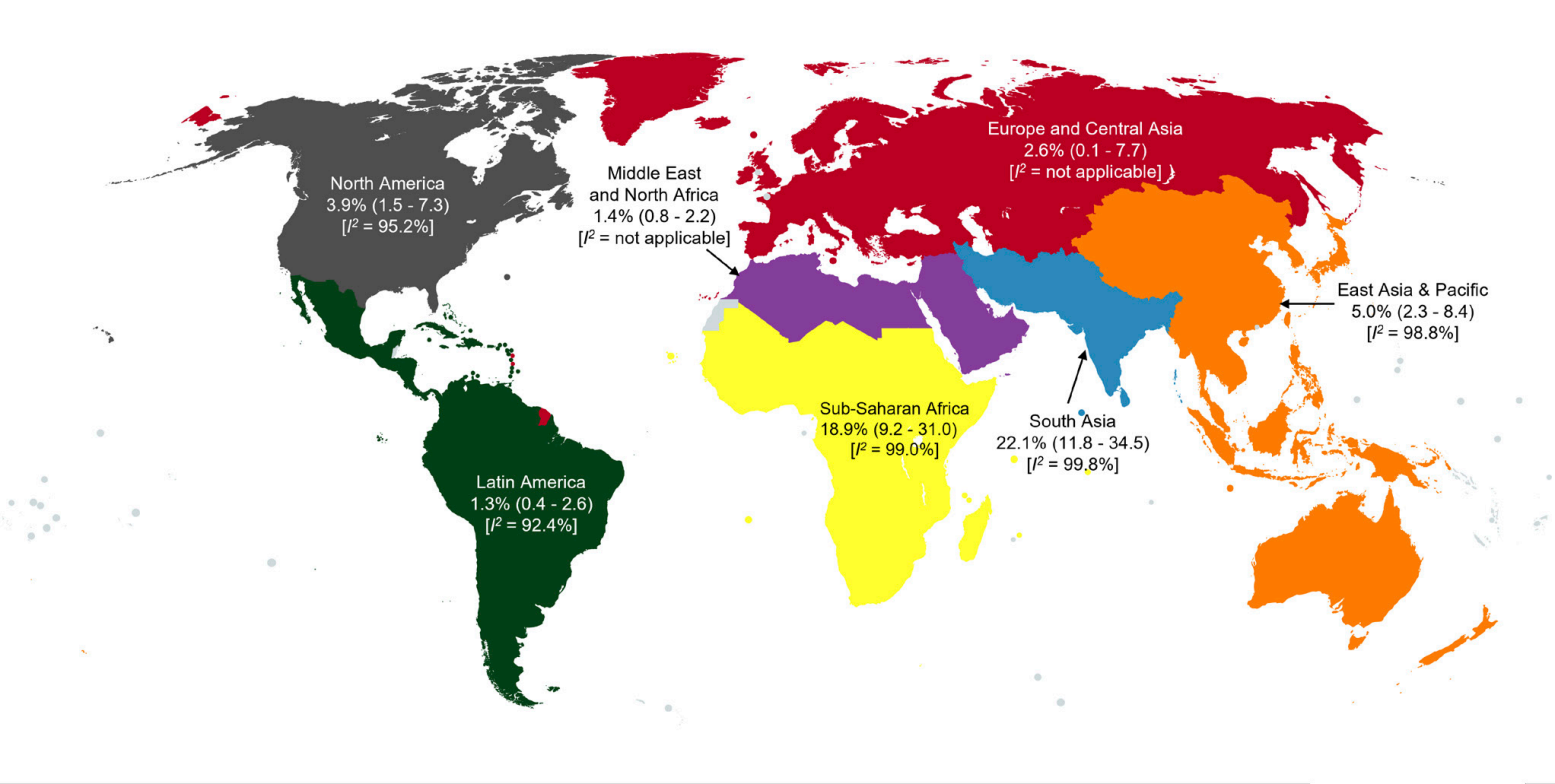
Geographically pooled prevalence of IDA among school-aged children in community settings.

**Figure 3:**
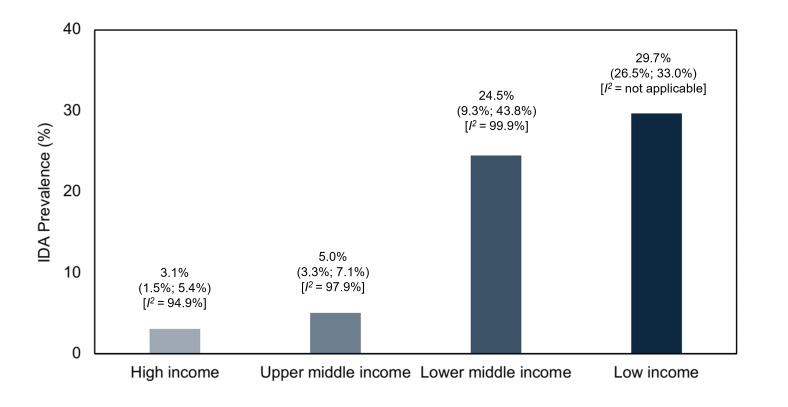
Prevalence of IDA among school-aged children classified by national economic status.

Due to significant heterogeneity, we performed subgroup analyses by continent and national economic status (**Figure 2**, **Figure 3**). The highest prevalence of IDA was noted in sub-Saharan Africa, at 21.9% (95% CI = 11.4–34.5, *I^2^* = 99.0%), followed by South Asia, at 15.8% (95% CI = 0.8–43.8, *I^2^* = 99.8%). Low-income and lower-middle income countries still had the highest prevalence of IDA, at 29.7% (95% CI = 26.5–33.0, *I^2^* = not applicable) and 24.5% (95% CI = 9.3–43.8, *I^2^* = 99.9%), respectively.

We further determined the pooled prevalence of IDA from both community and hospital-based settings (as opposed to community only) and by different diagnostic criteria (Table S5 in the **Online Supplementary Document**). The prevalence of IDA among children aged 5–12 years in both settings together was 8.7% (95% CI = 7.0–10.6, *I^2^* = 99.6%). The IDA prevalence according to the WHO criteria was 6.6% (95% CI = 5.3–8.1, *I^2^* = 99.5%), whereas it was 11.4% (95% CI = 4.8–20.2, *I^2^* = 99.3%) when using non-WHO criteria.

Lastly, we performed subgroup analyses by region and economic status for both community and hospital-based settings (as opposed to community only). We observed the highest prevalence of IDA in South Asia (19.6%; 95% CI = 5.0–40.3, *I^2^* = 99.8%) and sub-Saharan Africa (18.9%; 95% CI = 9.2–31.0, *I^2^* = 99.1%). The prevalence was greatest in low-income (29.7%; 95% CI = 26.5–33.0, *I^2^* = not applicable) and lower-middle income countries (22.5%; 95% CI = 10.1–38.0, *I^2^* = 99.8%). On the whole, the inclusion of hospital-based studies did not significantly affect the pooled global prevalence of IDA among children aged 5–12 years.

## DISCUSSION

Our study focuses on the global prevalence of IDA among children aged between 5–12 years. Following the WHO criteria, the public health significance of anaemia can be categorised as severe, moderate, and mild when the prevalence of anaemia was higher than 40%, 20–39.9%, and 5.0–19.9%, respectively [75]. Based on these thresholds, we classified the global prevalence of IDA among school-aged children of 9.4% a public health problem of mild significance. Sensitivity analysis incorporating both community-based and hospital-based settings showed a comparable prevalence to that observed in community-based studies only.

However, based on our subgroup analyses, we classified IDA as a moderate public health concern among children aged 5–12 years in certain geographic regions and income groups. The estimated prevalence of IDA was relatively higher in sub-Saharan Africa and South Asia than in Europe, Latin America, and North America. This finding aligns with the systematic and meta-analysis of the prevalence of IDA among children aged <5 years, where Asia and Africa had a higher prevalence compared to North America and Europe [7]. Based on national income, the estimated prevalence of IDA was higher in low and lower-middle-income countries than in other income groups. According to a WHO report, socioeconomic status strongly influences nutritional status and infection [76]. The population living in low- and lower-middle-income countries bear the greatest burden of anaemia, with iron deficiency and malaria being the predominant causes [76].

Due to differences in haemoglobin cutoffs, we performed subgroup analyses by IDA diagnostic criteria. According to the WHO criteria, anaemia is defined based on age- and gender- specific haemoglobin thresholds. For children, these thresholds are a concentration of <11.5 g/dL (ages 5–11 years) and <12.0 g/dL (ages 12–14 years), in combination with a serum ferritin level <15 μg/l. Accordingly, the estimated prevalence of IDA was 7.3% per the WHO and 12.4% per non-WHO criteria. Another important issue is the use of inflammation biomarkers, such as CRP, as an additional criterion to distinguish IDA from causes other than malnutrition. However, only three studies used CRP to confirm the diagnosis of IDA, preventing any sensitivity analysis in this sense. Therefore, the IDA prevalence observed in our study may reflect cases stemming from malnutrition and other aetiologies. Incorporating inflammation biomarkers should be considered in certain sub-populations with high rates of infection or related conditions, to better differentiate between IDA resulting from malnutrition and that associated with inflammatory causes. Notably, 8 out of 19 studies were conducted in the South Asia and sub-Saharan Africa regions, where the prevalence of IDA is higher compared to other regions. Furthermore, some regions, such as South Asia and sub-Saharan Africa, had wide CI, suggesting less stable prevalence estimates. Despite differences in the diagnostic criteria for IDA (*i.e.* WHO *vs*. non-WHO), the estimated global prevalence of IDA was classified as a mild public health concern. The diagnosis of IDA in all included studies was established based on laboratory results according to accepted diagnostic criteria, rather than solely on data from medical record documentation or ICD-10 coding alone, thereby enhancing the reliability of the collected data.

However, the prevalence of IDA remains a moderate public health issue in specific populations and geographical areas, including sub-Saharan Africa and South Asia, or low-income and lower-middle-income countries. Given IDA’s effect on cognitive impairment [5], there is a need for targeted public health strategies and interventions, such as continuous screening programmes, or supplementation and food fortification [13], that prioritise these high-burden areas and vulnerable populations and to effectively address the condition and its negative impacts on children's health, development, and educational outcomes. With global population ageing, supporting good health and well-being for children emerges as a priority for the next generation.

Our subgroup analysis by publication year, comparing studies published before 2015 to those published from 2015 onward, showed an approximately two-fold reduction in the prevalence of IDA among school-aged children (5–12 years), after adjusting for geography and diagnostic criteria. This overall significant reduction may be attributed to the 2011 WHO recommendation for intermittent iron supplementation in pre-school and school-aged children [13]. Nevertheless, the issue persists in sub-Saharan Africa and South Asia, where the prevalence remains above 15.0%.

Ours is the most up-to-date study on the global prevalence of IDA among school-aged populations. We assessed the quality of the included studies using Hoy’s risk of bias tool [18], which was specifically designed for population-based prevalence studies. Only 20% of the included studies had a moderate risk of bias, and none had a high risk of bias, lending credibility to the evidence in our study. The sensitivity analysis by risk of bias further showed that the prevalence of IDA regarding the low risk of bias was 7.3% – a result relatively similar to that our main analysis.

Some limitations to our study should be acknowledged. First, due to our inability to access high-quality translation tools or personnel, we included only English-language articles, which might have caused bias. To balance between the validity of the retrieved information and the comprehensiveness of evidence. we prioritised studies with clearly define diagnostic criteria, while also including across the countries and criteria to ensure global representation. Second, we had a limited number of studies for some subgroups, such as low-income countries or the Middle East & North Africa, which could affect the reliability of the evidence. This also highlights a need for further research in such context. Lastly, we noted substantial heterogeneity in all our analyses, which is not uncommon in meta-analyses of prevalence studies. We attempted to address this issue by employing a random effects model that considers between-study variances.

## CONCLUSIONS

IDA is a globally relevant public health issue among school-aged children (5–12 years), and can even be characterised as a moderately significant health concern in some regions. Our findings could guide the development of national detection strategies and health prevention programmes targeted at improving children's health and educational outcomes.

## Additional material


Online Supplementary Document
