# Supplementary material for: Global prevalence of iron deficiency anaemia among children aged 5–12 years: a systematic review and meta-analysis
Source: J Glob Health. 2026 Jan 23;16:04027. doi: 10.7189/jogh.16.04027 (PMC12828442; doi:10.7189/jogh.16.04027)
Supplement: Online Supplementary Document [file jogh-16-04027-s001.pdf]

Supplement to: Sukwuttichai P, Tidwong N, Chaipichit N, Dhippayom T, Dilokthornsakul W, Dilokthornsakul P. Global prevalence of iron deficiency anaemia among children aged 5–12 years: a systematic review and meta-analysis. J Glob Health. 2026;16:04027.

Online Supplementary Document

Table S1 Search strategy

1.1 PubMed

Search number	Query	Results
#1	Child* [Title/Abstract]	1,684,581
#2	Pre-adolscen* [Title/Abstract]	0
#3	Preadolescen* [Title/Abstract]	5,078
#4	Pre-teen* [Title/Abstract]	219
#5	Preteen* [Title/Abstract]	591
#6	School* [Title/Abstract]	347,724
#7	Elementary [Title/Abstract]	31,554
#8	Student* [Title/Abstract]	363,877
#9	Child [Mesh]	2,155,866
#10	Schools [Mesh]	146,277
#11	Students [Mesh]	168,146
#12	"Iron deficiency anemia" [Title/Abstract]	8,895
#13	"iron deficiency anaemia" [Title/Abstract]	2,830
#14	Anemia, iron-deficiency [Mesh]	11,724
#15	or/1-11	3,231,175
#16	or/12-14	17,948
#17	#15 AND #16	5,134
#18	NOT Clinical Trial [Publication Type]	4,583
#19	Limit to humans	4,079

1.2 Embase

Search number	Query	Results
#1	child*:ti,ab	2,177,700
#2	'pre-adolscen*':ti,ab	0
#3	preadolescen*:ti,ab	6,064
#4	'pre-teen*':ti,ab	316
#5	'preteen*':ti,ab	743
#6	school*:ti,ab	436,179
#7	elementary:ti,ab	35,064
#8	student*:ti,ab	474,179
#9	'child'/exp	3,370,172
#10	'schools'/exp	403,932
#11	'student'/exp	325,282
#12	'Iron deficiency anemia':ti,ab	14,082
#13	'iron deficiency anaemia':ti,ab	4,829
#14	'iron-deficiency anemia'/exp	35,600
#15	or/1-11	4,796,489
#16	or/12-14	38,596
#17	#15 AND #16	11,050
#18	#17 AND 'human'/de	10,142
#19	#18 AND 'article'/it	6,574
#20	#19 AND [embase]/lim NOT ([embase]/lim AND [medline]/lim)	1,558

1.3 CINAHL

Search number	Query	Results
#1	TI Child* OR AB Child*	593,193
#2	TI Pre-adolscen* OR AB Pre-adolscen*	0
#3	TI Preadolscen* OR AB Preadolscen*	1
#4	TI Pre-teen* OR AB Pre-teen*	128
#5	TI Preteen* OR AB Preteen*	245
#6	TI School* OR AB School*	172,064
#7	TI Elementary OR AB Elementary	8,947
#8	TI Student* OR AB Student*	212,138
#9	(MH “Child+”)	758,121
#10	(MH “School+”)	84,388
#11	(MH “Students+”)	174,558
#12	TI “Iron deficiency anemia” OR AB “Iron deficiency anemia”	2,074
#13	TI “Iron deficiency anaemia” OR AB “Iron deficiency anaemia”	686
#14	(MM “Anemia, Iron Deficiency”)	3,427
#15	or/1-11	1,289,120
#16	or/12-14	4,794
#17	#15 AND #16	1,779
#18	Limit to Humans	1,079
#19	Limit to Research Article	1,070

1.4 CINAHL

Search number	Query	Results
#1	TI Child* OR AB Child*	66,304
#2	TI Pre-adolscen* OR AB Pre-adolscen*	0
#3	TI Preadolscen* OR AB Preadolscen*	274
#4	TI Pre-teen* OR AB Pre-teen*	24
#5	TI Preteen* OR AB Preteen*	15
#6	TI School* OR AB School*	74,307

#7	TI Elementary OR AB Elementary	10,404
#8	TI Student* OR AB Student*	77,864
#9	KW Child*	19,439
#10	KW School*	17,932
#11	KW Student*	13,564
#12	TI "Iron deficiency anemia" OR AB "Iron deficiency anemia"	46
#13	TI "Iron deficiency anaemia" OR AB "Iron deficiency anaemia"	56
#14	KW TI "Iron deficiency anemia"	17
#15	or/1-11	180,149
#16	or/12-14	111
#17	#15 AND #16	40

Table S2 Protocol deviations from the registration in PROSPERO.

Protocol deviation issue	Original registered protocol	Revised protocol
Scope of the study	Studies conducted in Asia	Studies conducted worldwide
Population	Pre-school children aged <5 years	School-aged children aged 5 – 12 years
Search strategies	Included infant* and pre-school* and related terms	Changed to Pre-adolscen* and related terms (Table S1)

Table S3 The Quality of included studies using Hoy et al's Risk of Bias tool

List	Author	Year	Hoy et al's risk of bias criteria										Overall	
			Criteria 1	Criteria 2	Criteria 3	Criteria 4	Criteria 5	Criteria 6	Criteria 7	Criteria 8	Criteria 9	Criteria 10	Number of "YES"	Risk of bias
1	Abizari et al.	2017	N	N	N	N	Y	Y	Y	Y	Y	Y	6	Moderate
2	Afridi et al.	2017	N	N	N	N	Y	Y	Y	Y	Y	Y	6	Moderate
3	Al-Mekhlafi et al.	2008	N	N	Y	Y	Y	Y	Y	Y	Y	Y	8	Low
4	Andriastuti et al.	2020	N	N	N	N	Y	Y	Y	Y	Y	Y	6	Moderate
5	Baggett et al.	2006	N	N	Y	N	Y	Y	Y	Y	Y	Y	7	Low
6	Cardenas et al.	2005	Y	Y	Y	N	Y	Y	Y	Y	Y	N	8	Low
7	Cardoso et al	2012	N	Y	Y	Y	Y	Y	Y	Y	Y	Y	9	Low
8	Choi et al.	2003	N	N	N	N	Y	Y	Y	Y	Y	N	5	Moderate
9	Cojulun et al.	2015	N	Y	Y	Y	Y	Y	Y	Y	Y	Y	9	Low
10	Desalegn et al.	2014	N	Y	Y	Y	Y	Y	Y	Y	Y	Y	9	Low
11	Ferreira et al.	2007	N	Y	Y	Y	Y	Y	Y	Y	Y	N	8	Low
12	Fiorentino et al.	2013	N	Y		Y	Y	Y	Y	Y	Y	Y	8	Low
13	Gompakis et al.	2006	N	N	Y	Y	Y	Y	Y	Y	Y	Y	8	Low
14	Goosen et al	2022	N	N	N	N	Y	Y	Y	Y	Y	Y	6	Moderate
15	Gupta et al.	2017	Y	Y	Y	N	Y	Y	Y	Y	Y	Y	9	Low
16	Gwetu et al.	2019	N	N	Y	N	Y	Y	Y	Y	Y	Y	7	Low
17	Hlatswayo et al.	2016	N	N	Y	N	Y	Y	Y	Y	Y	Y	7	Low
18	Houghton et al.	2016	N	Y	Y	N	Y	Y	Y	Y	Y	Y	8	Low
19	Jaber et al.	2015	N	N	Y	N	Y	Y	Y	Y	Y	Y	7	Low
20	Khatiwada et al.	2016	N	N	Y	N	Y	Y	Y	Y	Y	Y	7	Low
21	Khemphet et al.	2022	N	N	N	N	Y	Y	Y	Y	Y	Y	6	Moderate
22	Kılınc ̧M, et al.	2002	N	Y	Y	N	Y	Y	Y	Y	Y	N	7	Low
23	Kuona P, et al.	2014	N	Y	Y	Y	Y	Y	Y	Y	Y	Y	9	Low
24	Liaqat A, et al.	2022	N	N	N	N	Y	Y	Y	Y	Y	Y	6	Moderate
25	López-Ruzafa et al.	2021	N	N	N	N	Y	Y	Y	Y	Y	Y	7	Low
26	Monárrez-Espino et al.	2004	N	Y	N	N	Y	Y	Y	Y	Y	N	6	Moderate
27	Ngui et al.	2012	N	Y	Y	Y	Y	Y	Y	Y	Y	Y	9	Low
28	Onabanjo et al.	2019	N	N	Y	Y	Y	Y	Y	Y	Y	Y	8	Low
29	Perignon et al.	2014	N	N	Y	Y	Y	Y	Y	Y	Y	Y	8	Low
30	Persson et al.	1999	N	N	N	Y	Y	Y	Y	Y	Y	Y	7	Low
31	Porniammongkol et al.	2011	N	N	N	Y	Y	Y	Y	Y	Y	Y	7	Low
32	Pouraram et al.	2018	Y	Y	Y	Y	Y	Y	Y	Y	Y	Y	10	Low
33	Rahman et al.	2015	Y	Y	Y	Y	Y	Y	Y	Y	Y	Y	10	Low
34	Robinson et al.	2018	N	Y	Y	Y	Y	Y	Y	Y	Y	Y	9	Low
35	Rohner et al.	2007	N	N	Y	Y	Y	Y	Y	Y	Y	Y	8	Low
36	Saengnipanthkul et al.	2022	N	Y	Y	Y	Y	Y	Y	Y	Y	N	8	Low
37	Sama et al.	2023	N	N	Y	Y	Y	Y	Y	Y	Y	Y	8	Low
38	Sarna et al.	2020	Y	Y	Y	Y	Y	Y	Y	Y	Y	Y	10	Low
39	Schieffer et al.	2017	N	Y	N	Y	Y	Y	Y	Y	Y	Y	8	Low
40	Shanita et al.	2018	Y	Y	Y	Y	Y	Y	Y	Y	Y	N	9	Low

List	Author	Year	Hoy et al's risk of bias criteria										Overall	
			Criteria 1	Criteria 2	Criteria 3	Criteria 4	Criteria 5	Criteria 6	Criteria 7	Criteria 8	Criteria 9	Criteria 10	Number of "YES"	Risk of bias
41	Spodaryk et al.	1999	N	N	N	Y	Y	Y	Y	Y	Y	N	6	Moderate
42	Sreekanth et al.	2021	N	N	N	Y	Y	Y	Y	Y	Y	Y	8	Low
43	Stellinga-Boelen et al.	2007	N	N	N	Y	Y	Y	Y	Y	Y	Y	7	Low
44	Stoltzfus et al.	1997	N	Y	Y	Y	Y	Y	Y	Y	Y	Y	9	Low
45	Syed et al.	2016	Y	Y	Y	N	Y	Y	Y	Y	Y	Y	9	Low
46	Tan et al.	2023	N	Y	Y	Y	Y	Y	Y	Y	Y	Y	9	Low
47	Tatala et al.	2004	N	N	N	N	Y	Y	Y	Y	Y	Y	6	Moderate
48	Teketelew et al.	2023	N	Y	Y	Y	Y	Y	Y	Y	Y	Y	9	Low
49	Turgut et al.	2007	N	N	N	N	Y	Y	Y	Y	Y	Y	6	Moderate
50	Valberg et al.	1976	Y	N	Y	Y	Y	Y	Y	Y	Y	Y	9	Low
51	Valencia et al.	1999	N	Y	N	N	Y	Y	Y	Y	Y	Y	8	Low
52	Vendt et al.	2011	N	Y	N	N	Y	Y	Y	Y	Y	Y	7	Low
53	Villalpando et al.	2015	Y	Y	N	N	Y	Y	Y	Y	Y	Y	8	Low
54	Yanola et al.	2014	N	Y	Y	Y	Y	Y	Y	Y	Y	Y	9	Low
55	Zheng et al.	2020	N	Y	Y	Y	Y	Y	Y	Y	Y	Y	9	Low

Criteria 1: Was the study's target population a close representation of the national population in relation to relevant variables?
Criteria 2: Was the sampling frame a true or close representation of the target population?
Criteria 3: Was some form of random selection used to select the sample OR was a census taken?
Criteria 4: Was the likelihood of nonresponse bias minimal?
Criteria 5: Were data collected directly from the subjects?
Criteria 6: Was an acceptable case definition used in the study?
Criteria 7: Was the study instrument that measured the parameter of interest shown to have validity and reliability?
Criteria 8: Was the same mode of data collection used for all subjects?
Criteria 9: Was the length of the shortest prevalence period for the parameter of interest appropriate?
Criteria 10: Were the numerator(s) and denominator(s) for the parameter of interest appropriate.
Y = Yes, N = No

Table S4 Prevalence of iron-deficiency anemia

List	Author	Year	Country of study	Economic status*	Setting	IDA		
						Number of participants (N)	Number of IDA (n)	Prevalence of IDA (95% CI)
1	Abizari et al.	2017	Ghana	Low middle income	Sub-Saharan Africa	224	102	0.46 (0.39-0.52)
2	Afridi et al.	2017	Pakistan	Low middle income	South Asia	700	241	0.34 (0.31-0.38)
3	Al-Mekhlafi et al.	2008	Malaysia	Upper middle income	East Asia &Pacific	241	82	0.34 (0.28-0.40)
4	Andriastuti et al.	2020	Indonesia	Upper middle income	East Asia &Pacific	45	5	0.11 (0.02-0.20)
5	Baggett et al.	2006	USA	High income	North America	683	53	0.08 (0.06-0.10)
6	Cardenas et al.	2005	USA	High income	North America	870	9	0.10 (0.08-0.12)
7	Cardoso et al	2012	Brazil	Upper middle income	Latin America	582	16	0.03 (0.01-0.04)
8	Choi et al.	2003	South Korea	High income	East Asia &Pacific	693	54	0.08 (0.06-0.10)
9	Cojulun et al.	2015	Kenya	Low middle income	Sub-Saharan Africa	191	83	0.43 (0.36-0.50)
10	Desalegn et al.	2014	Ethiopia	Low income	Sub-Saharan Africa	586	220	0.38 (0.34-0.41)
11	Ferreira et al.	2007	Brazil	Upper middle income	Latin America	80	8	0.10 (0.03-0.17)
12	Fiorentino et al.	2013	Senegal	Low middle income	Sub-Saharan Africa	279	28	0.10 (0.07-0.14)
13	Gompakis et al.	2006	Greece	High income	Europe & Central Asia	865	3	0.00 (0.00-0.01)
14	Goosen et al	2022	South Africa	Upper middle income	Sub-Saharan Africa	291	22	0.08 (0.05-0.11)
15	Gupta et al.	2017	India	Low middle income	South Asia	132	29	0.22 (0.15-0.29)
16	Gwetu et al.	2019	South Africa	Upper middle income	Sub-Saharan Africa	184	9	0.05 (0.02-0.08)
17	Hlatswayo et al.	2016	South Africa	Upper middle income	Sub-Saharan Africa	194	19	0.10 (0.06-0.14)

List	Author	Year	Country of study	Economic status*	Setting	IDA		
						Number of participants (N)	Number of IDA (n)	Prevalence of IDA (95% CI)
18	Houghton et al.	2016	New Zealand	High income	East Asia &Pacific	503	0	-
19	Jaber et al.	2015	Israel	High income	Middle East & North Africa	693	15	0.02 (0.01-0.03)
20	Khatiwada et al.	2016	Nepal	Low middle income	South Asia	316	99	0.31 (0.26-0.36)
21	Khemphet et al.	2022	Thailand	Upper middle income	East Asia &Pacific	99	3	0.03 (0.00-0.06)
22	Kılınc ̧M, et al.	2002	Turkey	Upper middle income	Europe & Central Asia	295	37	0.13 (0.09-0.16)
23	Kuona P, et al.	2014	Zimbabwe	Low middle income	Sub-Saharan Africa	318	7	0.02 (0.01-0.04)
24	Liaqat A, et al.	2022	Pakistan	Low middle income	South Asia	76	20	0.26 (0.16-0.36)
25	López-Ruzafa et al.	2021	Spain	High income	Europe & Central Asia	491	0	-
26	Monárrez-Espino et al.	2004	Mexico	Upper middle income	Latin America	75	7	0.09 (0.03-0.16)
27	Ngui et al.	2012	Malaysia	Upper middle income	East Asia &Pacific	520	85	0.16 (0.13-0.20)
28	Onabanjo et al.	2019	South Africa	Upper middle income	Sub-Saharan Africa	556	31	0.06 (0.04-0.07)
29	Perignon et al.	2014	Cambodia	Low middle income	East Asia &Pacific	2443	238	0.10 (0.09-0.11)
30	Persson et al.	1999	Bangladesh	Low middle income	South Asia	164	23	0.14 (0.09-0.19)
31	Porniammongkol et al.	2011	Thailand	Upper middle income	East Asia &Pacific	34	2	0.06 (0.00-0.14)
32	Pouraram et al.	2018	Iran	Upper middle income	Middle East & North Africa	8500	425	0.05 (0.05-0.05)
33	Rahman et al.	2015	Bangladesh	Low middle income	South Asia	94400	944	0.01 (0.02-0.14)

List	Author	Year	Country of study	Economic status*	Setting	IDA		
						Number of participants (N)	Number of IDA (n)	Prevalence of IDA (95% CI)
34	Robinson et al.	2018	Colombia	Upper middle income	Latin America	1042	0	-
35	Rohner et al.	2007	Coˆte d'Ivoire	Low middle income	Sub-Saharan Africa	281	101	0.36 (0.30-0.42)
36	Saengnipanthkul et al.	2022	Thailand	Upper middle income	East Asia &Pacific	2066184	13025	0.01 (0.01-0.01)
37	Sama et al.	2023	Cameroon	Low middle income	Sub-Saharan Africa	154	28	0.18 (0.12-0.24)
38	Sarna et al.	2020	India	Low middle income	South Asia	2064	578	0.28 (0.26-0.30)
39	Schieffer et al.	2017	USA	High income	North America	6978	129	0.02 (0.02-0.02)
40	Shanita et al.	2018	Malaysia	Upper middle income	East Asia &Pacific	544	2	0.00 (0.00-0.01)
41	Spodaryk et al.	1999	Poland	High income	Europe & Central Asia	188	12	0.06 (0.03-0.10)
42	Sreekanth et al.	2021	India	Low middle income	South Asia	52	7	0.13 (0.04-0.23)
43	Stellinga-Boelen et al.	2007	Netherlands	High income	Europe & Central Asia	71	3	0.04 (0.00-0.09)
44	Stoltzfus et al.	1997	Tanzania	Low middle income	Sub-Saharan Africa	3254	1673	0.51 (0.50-0.53)
45	Syed et al.	2016	Mexico, Colombia	Upper middle income	Latin America	11933	180	0.02 (0.01-0.02)
46	Tan et al.	2023	Malaysia	Upper middle income	East Asia &Pacific	776	47	0.06 (0.04-0.08)
47	Tatala et al.	2004	Tanzania	Low middle income	Sub-Saharan Africa	80	25	0.31 (0.21-0.41)
48	Teketelew et al.	2023	Ethiopia	Low income	Sub-Saharan Africa	187	18	0.10 (0.05-0.14)
49	Turgut et al.	2007	Turkey	Upper middle income	Sub-Saharan Africa	256	23	0.09 (0.05-0.12)
50	Valberg et al.	1976	Canada	High income	North America	117	5	0.04 (0.01-0.08)

List	Author	Year	Country of study	Economic status*	Setting	IDA		
						Number of participants (N)	Number of IDA (n)	Prevalence of IDA (95% CI)
51	Valencia et al.	1999	Mexico	Upper middle income	Latin America	296	2	0.01 (0.00-0.02)
52	Vendt et al.	2011	Estonia	High income	Europe & Central Asia	135	1	0.01 (0.00-0.02)
53	Villalpando et al.	2015	Mexico	Upper middle income	Latin America	4320	60	0.01 (0.01-0.02)
54	Yanola et al.	2014	Thailand	Upper middle income	East Asia &Pacific	130	1	0.01 (0.00-0.02)
55	Zheng et al.	2020	China	Upper middle income	East Asia &Pacific	5295	32	0.01 (0.00-0.01)

*Economic status based on the World Bank 2024

CI; confidence interval, IDA; iron-deficiency anemia

Table S5 A sensitivity analysis by pooling both community and hospital settings.

	**Prevalence (%) (95% confidence interval)**	**I^2(%)**
**Overall (both community and hospital settings)**	8.7 (7.0; 10.6)	99.6
**Diagnostic criteria of IDA**		
WHO criteria	6.6 (5.3; 8.1)	99.5
Non-WHO criteria	11.4 (4.8; 20.2)	99.3
**Regions**		
North America	3.2 (1.0; 6.3)	95.2
Latin America	1.6 (0.7; 2.8)	92.4
Sub-Saharan Africa	18.9 (9.2; 31.0)	99.1
East Asia & Pacific	5.0 (2.3; 8.4)	99.1
South Asia	19.6 (5.0; 40.3)	99.8
Middle East & North Africa	4.7 (4.3; 5.2)	NA
Europe & Central Asia	2.6 (0.1; 7.7)	95.8
**National Economic Status**		
High income countries	2.1 (0.1; 3.8)	94.8%
Upper-middle income countries	4.7 (3.3; 6.3)	98.8%
Lower-middle income countries	22.5 (10.1; 38.0)	99.8%
Low-income countries	29.7 (26.5; 33.0)	N/A

N/A; not applicable, IDA; Iron-deficiency anemia, WHO; World Health Organization,
Diagnosis criteria of IDA based on Hemoglobin (g/dl) and ferritin (ug/l): WHO defines IDA by Hemoglobin < 11.5 g/dl (age 5-11 years)
or <12 g/dl (age 12-14 year) and ferritin <15 (ug/l).

Table S6: A sensitivity analysis by year of the published studies

	**Studies published before 2015**	**Studies published in 2015 onward**
**Overall**	11.7% (95%CI: 5.5; 19.7, I^2 = 99.3%)	7.0% (95%CI: 4.7; 9.6, I^2 = 99.4%)
**Diagnostic criteria of IDA***		
Non-WHO	10.6% (95%CI: 2.3; 23.6, I^2 = 99.5%)	17.9% (95%CI: 6.1; 34.0, I^2 = 97.9%)
WHO	13.7% (95%CI: 6.7; 22.5, I^2 = 98.1%)	4.6% (95%CI: 2.7; 6.9, I^2 = 99.4%)
**Regions**		
North America	3.8% (95%CI: 0.3; 10.5, I^2: N/A)	1.8% (95%CI: 1.6; 2.2, I^2: N/A)
Latin America	4.2% (95%CI: 1.1; 9.1, I^2 = 86.5%)	0.8% (95%CI: 0.2; 1.7, I^2: N/A)
Sub-Saharan Africa	27.7% (95%CI: 13.6; 44.5, I^2 = 98.9%)	17.2% (95%CI: 6.7; 31.3, I^2 = 97.8%)
East Asia & Pacific	11.0% (95%CI: 5.5; 17.9, I^2 = 96.2%)	1.6% (95%CI: 0.1; 4.6, I^2 = 96.2%)
South Asia	14.0% (95%CI: 9.5; 20.2, I^2: N/A)	16.4% (95%CI: 0.2; 50.2, I^2: N/A)
Middle East & North Africa	No included study	7.3% (95%CI: 5.0; 10.0, I2 = 99.4%)
Europe & Central Asia	7.9% (95%CI: 3.7; 13.3, I^2: N/A)	No included study
**National economic status**		
High-income countries	4.9% (95%CI: 2.0; 8.9, I^2 = 92.8%)	1.0% (95%CI: 0.1; 2.7, I^2: N/A)
Upper-middle income countries	8.4% (95%CI: 3.3; 15.4, I^2 = 96.3%)	2.8% (95%CI: 1.4; 4.5, I^2 = 98.1%)
Lower-middle income countries	23.8% (95%CI: 7.0; 46.6, I^2 = 99.6%)	25.1% (95%CI: 6.0; 51.4, I^2 = 99.8%)
Low-income countries	37.5% (95%CI: 33.7; 41.5, I^2: N/A)	9.6% (95%CI: 6.2; 14.7, I^2: N/A)

*Diagnosis criteria of IDA based on Hemoglobin (g/dl) and ferritin (ug/l): WHO defines IDA by Hemoglobin < 11.5 g/dl (age 5-11 years) or < 12 g/dl (age 12-14 year) and ferritin <15 (ug/l).

N/A; not applicable, IDA; Iron-deficiency anemia

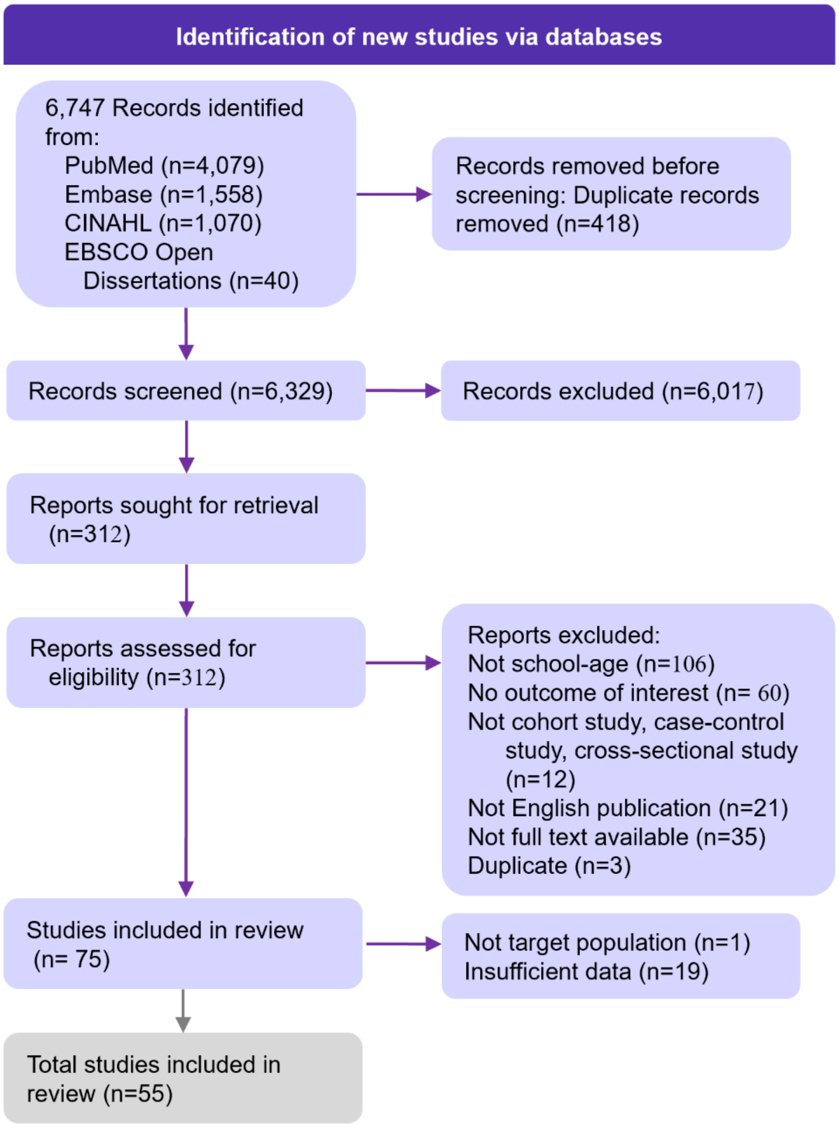

Figure S1 PRISMA flow diagram of selected articles
